# A genome‐wide investigation of the worldwide invader *Sargassum muticum* shows high success albeit (almost) no genetic diversity

**DOI:** 10.1111/eva.12837

**Published:** 2019-08-14

**Authors:** Sabrina Le Cam, Claire Daguin‐Thiébaut, Sarah Bouchemousse, Aschwin H. Engelen, Nova Mieszkowska, Frédérique Viard

**Affiliations:** ^1^ Station Biologique de Roscoff, CNRS Laboratory Adaptation & Diversity in Marine Environments (UMR 7144 CNRS SU), Sorbonne Université Roscoff France; ^2^ Center for Marine Sciences (CCMAR) University of Algarve Faro Portugal; ^3^ Marine Biological Association of the U.K. (MBA) Plymouth UK; ^4^ School of Environmental Sciences University of Liverpool Liverpool UK

**Keywords:** biological invasion, non‐native species, population genomics, RAD sequencing, seaweed, selfing

## Abstract

Twenty years of genetic studies of marine invaders have shown that successful invaders are often characterized by native and introduced populations displaying similar levels of genetic diversity. This pattern is presumably due to high propagule pressure and repeated introductions. The opposite pattern is reported in this study of the brown seaweed, *Sargassum muticum*, an emblematic species for circumglobal invasions. Albeit demonstrating polymorphism in the native range, microsatellites failed to detect any genetic variation over 1,269 individuals sampled from 46 locations over the Pacific–Atlantic introduction range. Single‐nucleotide polymorphisms (SNPs) obtained from ddRAD sequencing revealed some genetic variation, but confirmed severe founder events in both the Pacific and Atlantic introduction ranges. Our study thus exemplifies the need for extreme caution in interpreting neutral genetic diversity as a proxy for invasive potential. Our results confirm a previously hypothesized transoceanic secondary introduction from NE Pacific to Europe. However, the SNP panel unexpectedly revealed two additional distinct genetic origins of introductions. Also, conversely to scenarios based on historical records, southern rather than northern NE Pacific populations could have seeded most of the European populations. Finally, the most recently introduced populations showed the lowest selfing rates, suggesting higher levels of recombination might be beneficial at the early stage of the introduction process (i.e., facilitating evolutionary novelties), whereas uniparental reproduction might be favored later in sustainably established populations (i.e., sustaining local adaptation).

## 1 INTRODUCTION

Biological introductions have been reported at expanding scales and increasing rates since the 20th century in all ecosystems. Despite a long‐standing scientific interest in the understanding of what makes a species successful once introduced in a new range, this is still an unresolved and pressing issue (e.g., Briski et al., 2018; Pearson, Ortega, Eren, & Hierro, 2018; Seebens et al., 2017). A positive link between genetic diversity and invasion success has been hypothesized (Bock et al., 2015) as, consistently across taxa and environments, many successfully introduced species are not genetically depauperate (Bock et al., 2015; Roman & Darling, 2007; Viard, David, & Darling, 2016). This link is well illustrated in marine species introduced in European seas (Rius, Turon, Bernardi, Volckaert, & Viard, 2015): 74% of the studied European nonindigenous species (NIS) display similar or even higher levels of genetic diversity in introduced compared with native populations. The increased genetic diversity was often found associated with multiple introductions from different origins.

In the coastal marine environment, the number of introductions has dramatically increased over the past century with the generalization of transoceanic shipping, intra‐coastal boating, and aquaculture (Molnar, Gamboa, Revenga, & Spalding, 2008; Nunes, Katsanevakis, Zenetos, & Cardoso, 2014). In this context, marine introductions are often characterized by polyvectism (each pathway can act as a consecutive relay), repeated introductions, and overall high propagule pressure. The subsequent overall increase in the genetic variance of introduced populations, as compared to populations of the native range, might thus result in an increased standing genetic variation on which selection might operate or admixture between distinct genetic lineages creating evolutionary novelties. Both these processes presumably enhance the introduction success in marine environments (Rius & Darling, 2014; Rius et al., 2015; Viard et al., 2016).

**Figure 1:**
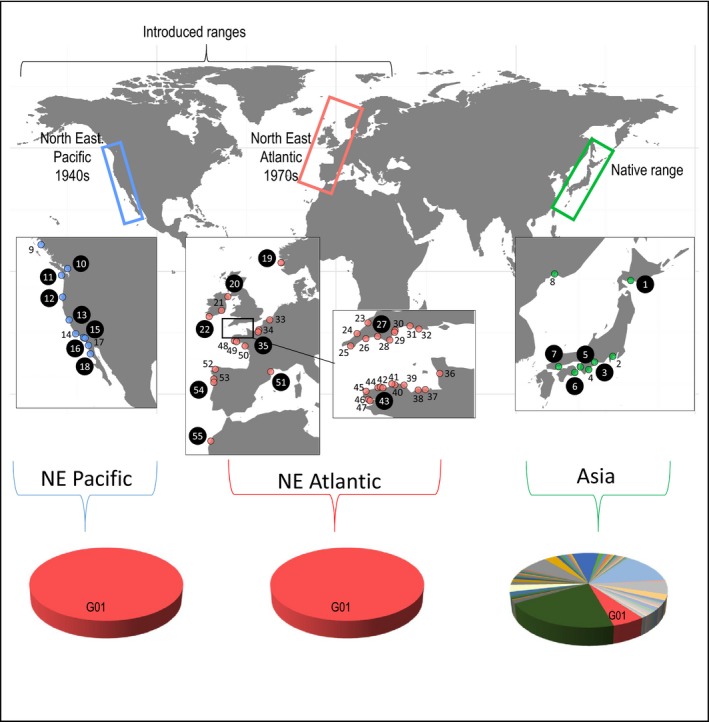
Study areas, sampling localities, and microsatellite genetic diversity of *Sargassum muticum*. Three geographic areas were sampled: 1) the native range in Asia and 2) two introduction ranges in the NE Pacific and NE Atlantic. All localities were analyzed with 14 microsatellite loci: eight sites in the native range, nine sites in the NE Pacific, and 37 sites in the NE Atlantic. The relative abundance of each of the 60 multi‐locus genotypes in the three areas is indicated. The localities also analyzed with ddRAD sequencing are highlighted by numbers in large black circles. Details about sample size and localities are provided in Table S1

In this study, we examined whether one of the most successful invasive seaweeds, *Sargassum muticum* (Yendo) Fensholt 1955, also displays the typical high genetic diversity reported in other successfully introduced marine taxa. So far, most marine species studied are animals (e.g., 86% of the studies examined in Rius et al. (2015)), and yet hundreds of introduced seaweeds have been reported (346 species reported as invasive; Thomsen, Wernberg, South, & Schiel, 2016). Among them, *S. muticum* is an emblematic species for circumglobal invasions and its native distribution and history of introduction are well documented (reviewed in Engelen et al., 2015). This seaweed, native to Asia, was first reported outside its native range in the 1940s in the North East Pacific in the Strait of Georgia, British Columbia. A second wave of introduction started in the 1970s in the North East Atlantic, with a first report on the Isle of Wight, United Kingdom. *S. muticum* subsequently spread rapidly along the NE Pacific and NE Atlantic coasts, in both southward and northward directions. Its present‐day introduced range spans an extremely large latitudinal gradient: from Alaska to Mexico in the NE Pacific and from Norway to Morocco in the NE Atlantic (Figure 1). In the NE Atlantic, the species is particularly well established, forming abundant populations, and the expansion is still in progress with new populations established in 2012 in Morocco (Sabour, Reani, El Magouri, & Haroun, 2013).

The worldwide expansion of *S. muticum* has mostly been explained by accidental introductions through deliberate imports of oysters on which the species grows (Engelen et al., 2015). Oyster culture has been recognized as one of the major vectors of introductions worldwide (Haupt, Griffiths, & Robinson, 2012; Wolff & Reise, 2002), because of repeated intra‐ and inter‐continental translocations of several oyster species outside of their native range. These movements are illustrated with the imports of *Crassostrea virginica,* from the NW to the NE Atlantic in the 1800s, and of the Pacific oyster *Crassostrea (Magallana) gigas* from Asia to North America and Europe which started in the 1940s. They can be responsible for both primary introductions (i.e., from the native range to a new area) and secondary introductions (i.e., from an introduced range to a new one, as exemplified by the introduction of the gastropod *Ocinebrellus inornatus* in France, (Martel, Viard, Bourguet, & Garcia‐Meunier, 2004)). For species accidentally introduced with oysters, such as *Crepidula fornicata* (Riquet, Daguin‐Thiébaut, Ballenghien, Bierne, & Viard, 2013), *Ocinebrellus inornatus* (Pante et al., 2015; named as *Ocenebra inornata* in this paper), or *Polysiphonia morrowii* (Geoffroy et al., 2016), important genetic diversity has been documented in introduced populations. As such, we hypothesized that the genetic diversity of *S. muticum* in its American and European introduced ranges should be high (i.e., close to what is observed in its native range). A previous study based on mitochondrial sequencing (Cheang et al., 2010) failed to elucidate the introduction history or evaluate the extent of founder events due to very low marker resolution. In this study, we developed nuclear polymorphic markers and carried out extensive sampling of 1,500 individuals from American, European, and native Asian populations to examine the introduction history, and more specifically the changes in genetic diversity accompanying the introduction process at a global scale.

Besides multiple origins and propagule pressure, other key factors in especially large scale and fast spreading successful introductions are facilitating life‐history traits, such as microscopic, dispersive and/or dormant stages, high fecundity, and uniparental reproduction. These traits are all likely to decrease the effect of demographic stochasticity in the early stage of introduction and/or at the front of the expansion wave. Like in terrestrial plants, seaweeds often display uniparental reproduction, either through clonal reproduction or selfing. In light of Baker's law, such reproductive traits are expected to facilitate the colonization of new habitats (Pannell et al., 2015). In the red seaweed *Gracilaria vermiculophylla*, Krueger‐Hadfield et al. (2016) highlighted a shift from sexual to asexual reproduction in introduced populations and, in the kelp *Undaria pinnatifida*, Guzinski, Ballenghien, Daguin‐Thiébaut, Lévêque, and Viard (2018) observed particularly high selfing rates in populations colonizing marinas, known to be disturbed habitats. Partial selfing is undoubtedly one of the life‐history traits of *Sargassum muticum* likely to explain its rapid spread and fast local expansion in a novel environment (Engelen et al., 2015). Consequently, compared with other marine introduced species, *S. muticum* could display more reduction in genetic diversity in its introduction range due to this reproductive property. We thus also compared selfing rates in native and introduced populations.

Based on previous studies of marine invaders, in particular species found both in N America and Europe, and previous studies on *S. muticum* (Cheang et al., 2010; Engelen et al., 2015), our expectations were (a) the absence of founder events in the primary introduction range (i.e., in NE Pacific), due to high propagule pressure and repeated accidental introductions with oyster imports since the 1940s, (b) a secondary introduction from the NE Pacific to Europe, through reported imports of oysters between the NE Pacific (more specifically the Puget Sound) and Europe (notably France) in the mid‐20th century, as reported in other NIS, and (c) increased selfing rates in introduced as compared with native populations, as a facilitating trait for the establishment of new populations in the new range. To ascertain these hypotheses, we first developed microsatellite markers as none were available for the study species. Microsatellites are easy to use for genotyping vast numbers of individuals (here we targeted ca. 1,500 individuals) and have been shown to be useful in analyzing introduction patterns and processes in marine organisms, including seaweeds. However, for detailed investigation, we had to develop a second marker panel, with SNPs obtained from ddRAD sequencing, a technique providing a reduced representation of the genome.

## 2 MATERIAL AND METHODS

### 2.1 Sampling

A total of 1,500 specimens from 55 locations (Figure 1, Table S1) were studied, with a focus in the NE Atlantic (1,083 individuals, 37 locations) where the species is still spreading (e.g., first observation in Morocco in 2012). For comparisons, eight populations from the native range (231 individuals) with most populations from Japan from where the introduced individuals in the NE Pacific have been presumed to come from (Cheang et al., 2010; Engelen et al., 2015) and ten from the American introduction range (188 individuals) were analyzed. For the NE Atlantic and NE Pacific, particular care was paid to obtaining samples representative of the full latitudinal range colonized by the species, thus across contrasting environments. In the field, for each specimen, a fragment of the thallus was cut and preserved in silica gel for subsequent genetic analyses. Individuals from 21 sites were subsampled to perform ddRAD sequencing (see below), from both the native and introduced ranges: five sites (*N* = 78) across Japan and 16 sites across the two areas of introduction, with nine sites in the NE Atlantic (*N* = 138) and seven sites (*N* = 87) in the NE Pacific (Figure 1, Table S1). For ddRAD sequencing, we selected populations encompassing the whole latitudinal range in the two areas, except Alaska for the NE Pacific for which no individuals gave satisfactory results.

### 2.2 Microsatellite isolation and genotyping

Microsatellite loci were isolated from 1 µg of *S. muticum* genomic DNA, obtained from six individuals sampled in the NE Atlantic (i.e., introduced range), by the Genoscreen Company (Lille, France, http://www.genoscreen.fr) according to a procedure coupling microsatellite enrichment and pyrosequencing with 454 GS‐FLX titanium technology (Malausa et al., 2011). The development of the microsatellite markers (including polymorphism screening) was then pursued using 14 individuals sampled both in the native (Japan and Korea) and introduced (Spain, UK, France) ranges, to avoid ascertainment biases, in particular the risks to select markers polymorphic only in the native populations. Among the 138 primer pairs primarily tested for amplification using a subset of 14 Asian and European individuals, 32 loci did not amplify, 19 loci did not yield reliable amplification patterns, and 29 were monomorphic, resulting in 14 loci (Table S2) selected for routine genotyping.

For routine genotyping, DNA of each individual was extracted from 5 to 10 mg of dried tissue and eluted in 120 µl of elution buffer using the Nucleospin 96 Plant kit (Macherey‐Nagel) according to the manufacturer's instructions. For multiplex PCR amplifications (Table S2), 2 µl of 1:50 diluted DNA template was used in a 10 µl final volume containing 0.25 mM of each dNTP, 2.25 mM of magnesium chloride, 0.1 mg/ml of bovine serum albumin, and 0.3 U of Thermoprime plus DNA polymerase (Thermo ABgene). Final primer concentrations varied from 33 nM (for some dye‐labeled forward primers) to 234 nM (for all reverse primers) according to loci. A unique touchdown PCR program was used: 5 min at 95°C followed by 10 cycles with 30 s at 95°C, 30 s at 62°C and then decreasing by 1°C per cycle to 52°C, 50 s at 72°C, and then 25 cycles at 95°C for 30 s, 52°C for 30 s, 72°C for 50 s, with a final extension step of 15 min at 72°C. Two µl of PCR products was added to 10 µl of Hi‐Di formamide (Applied Biosystems) containing a size marker (Mauger, Couceiro, & Valero, 2012) and electrophoresed in a 3130XL genetic analyser (Applied Biosystems). Allele sizing was performed using the Genemapper v4.0 software (Applied Biosystems).

### 2.3 RAD‐seq library construction and SNP calling

Double‐digest RAD‐seq individual libraries were produced following Brelsford, Dufresnes, and Perrin (2016). Briefly, genomic DNA extracts (see above) were quantified using Quantit Picogreen dsDNA Assay Kits (Life Technologies). For each individual, 100 ng of genomic DNA was digested with PstI and MseI restriction enzymes (New England Biolabs). Barcoded adaptors were ligated to the digested DNA fragments and purified using AMPure XP Beads (Beckman Coulter). Two microliters of the purified template was used for enrichment and Illumina indexing by PCR using Q5 hot start DNA polymerase (New England Biolabs), with 12 cycles and four replicates per samples. The 303 samples from 21 sampling sites were then pooled in equal proportions in three separate libraries. Individuals from each of the three studied areas (i.e., Asia, NE Pacific and NE Atlantic) were distributed in each library to reduce potential biases due to the wet laboratory procedure during the library development on subsequent analyses (O'Leary, Puritz, Willis, Hollenbeck, & Portnoy, 2018). A 250–550 base pair size selection of fragments was performed using a 1.5% agarose cassette in a pippin prep equipment (Sage Science). A pool of 12 replicates (wet lab replicates and sequencing replicates) were added to each library, so in total 339 individually tagged DNAs were sequenced. To increase genotyping confidence by high sequencing coverage, each library was sequenced twice, on two lanes of an Illumina Hiseq 2,500 generating 125 bp single‐end reads at Eurofins.

A total of 1.16 billion reads were obtained across the three libraries. Raw sequences were quality checked and demultiplexed using the *process_radtags* module of Stacks v1.32 (Catchen, Hohenlohe, Bassham, Amores, & Cresko, 2013). We used the Stacks denovo_map.pl pipeline for locus assembly and SNP calling. Replicates were first analyzed independently to determine the optimal denovo parameters. As recommended in Paris, Stevens, and Catchen (2017), multiple stack parameter was tested to determine the optimal de novo parameters. A set of five replicates were analyzed using the methodology of Mastretta‐Yanes et al. (2015) to minimize the genotyping errors. A de novo analysis was then performed on 303 samples with the following parameters: ‐m (minimum stack depth) 10, ‐M (maximum number of mismatches between alleles at a locus within an individual) 2, ‐*n* (maximum number of mismatches between alleles at a locus across all individuals) 2, a maximum of two stacks per locus, and SNP calling bounded model type (high bound 0.01). Catalog loci were filtered according to mean log likelihood scores >−30 using the *rxstacks* module and according to their missing rate (genotyped in minimum 80% of the populations and 50% of individuals per population). Haplotype‐based genotypes (RAD_loci) were exported from Stacks. Repetitive sequences can be confounded with alleles in denovo analyses of RAD‐seq data, leading to an uneven coverage among alleles in a given locus. The mean allelic coverage ratio was calculated for all heterozygote RAD‐seq loci and tested against the expected coverage ratio using Student's *t* test. The distribution of *p*‐values was used to set a threshold for excluding RAD‐seq loci with imbalanced allelic coverage. Finally, loci with a minor allele frequency (MAF) lower than 0.01 and observed heterozygosity higher than 0.5 were filtered out. The final dataset was made of 8,788 polymorphic loci in a sample set of 300 individuals from 21 sampling sites, with 20% missing data across the whole dataset. In addition, for selfing rates, multi‐locus genotypes (MLGs), and genetic structure analyses (see below), we used an even more conservative dataset made of 256 individuals, with <10% of missing data. In this conservative dataset, only one individual from the San Francisco—Pier 39 (no. 13) locality was retained and used in individual‐based analyses (i.e., MLG inferences, Bayesian clustering and Principal Component Analyses, see below).

### 2.4 Genetic diversity and selfing rate estimation

Estimates of gene diversity, observed heterozygosity, and mean number of alleles per locus were calculated using GeneClass v2.0 (Piry et al., 2004). Weir and Cockerham (1984) *f* and *theta* estimates of *F*_is_ and *F*_st_ indices were estimated with Genepop v4.2.2 (Rousset, 2008) and tested using G‐exact tests (MCMC parameters: 10,000 dememorization, 100 batches, 5,000 iterations). Pairs of loci in linkage disequilibrium were determined for each pair of microsatellite loci, and for each population, and the null hypothesis of independence of genotypes was tested with a G test implemented in Genepop.

The population selfing rate (*s*) was estimated from the distributions of multi‐locus heterozygosity in each population (David, Pujol, Viard, Castella, & Goudet, 2007) with *g*2, an estimator of the correlation of heterozygosity across loci, computed using RMES (David et al., 2007) for microsatellite loci and inbreedR (Stoffel et al., 2016) for RAD‐seq loci. For both markers, the selfing rate (*s*_g2_) was computed following the equation 9 in David et al. (2007).

Effective recombination rates are expected to be low in the study species considering the extremely high estimated selfing rates in the study populations. We thus examined the diversity and distribution of the multi‐locus genotypes (MLGs) at microsatellites and RAD‐seq loci. For the 14 microsatellites, the analysis was straightforward and MLGs were directly compiled through the dataset and the number of private MLG per population counted. Genotypic diversity was computed by N/(N−1) (1−∑p_i_^2^) where p_i_ is the genotypic frequency of the ith genotype. For RAD‐seq loci, MLGs were clustered into multi‐locus lineages based on the distribution of pairwise genetic distance using the R package poppr (Kamvar, Tabima, & Grünwald, 2014). A cutoff threshold was defined based on the “farthest neighbour” algorithm to collapse genotypes into the same multi‐locus lineage.

### 2.5 Spatial genetic structure analyses

Genetic structure was estimated with two approaches: a non‐model‐based approach using a principal component analysis (PCA), a multivariate method implemented in the package adegenet 1.4–2 (Jombart & Ahmed, 2011) and a model‐based clustering algorithm implemented in the InStruct software (Gao, Williamson, & Bustamante, 2007) to test individual admixture proportions and the correspondence of genetic clusters with the sampled populations. InStruct is extending the model implemented in STRUCTURE (Pritchard, Stephens, & Donnelly, 2000) by releasing the assumption of Hardy–Weinberg equilibrium within clusters, mandatory here considering the variable and sometimes high selfing rates observed in the study populations. InStruct simultaneously estimates selfing rates and assigns individuals to one of *K* genetic clusters without a priori grouping of the individuals in populations (five independent runs for each *K*, 50,000 burn‐in steps, 100,000 MCMC steps). The best value for the number of cluster *K* was determined by the deviance information criteria (DIC). Graphical output was obtained using CLUMPAK.

## 3 RESULTS

### 3.1 Microsatellite diversity

In the native range, the 14 microsatellite loci displayed two to three alleles each, and moderate genetic diversity within populations (e.g., He < 0.3; Table S3) together with high genetic structure among populations (e.g., global Fst = 0.72, *P*_exact test_ < 10^–5^; Table S4 for population pairwise values). Sixty multi‐locus genotypes (MLGs) were detected among the 220 individuals screened in the native range (Figure 1), most of them private to one population (Table S3). Estimated selfing rates were very high (above 0.76 in four native populations, Table S2). The selfing rate was, however, not different from zero in three populations, namely Oshima, Kashikojima, and Inhoshima (no. 2, 3, and 4 in Figure 1, respectively; Table S3).

In sharp contrast with the native range, no genetic diversity was observed across the entire introduced range: all of the 1,269 individuals shared the same MLG (G01 in Figure 1, Table S3). This unique MLG was detected in only one population in the native range (no. 6 (Anan) in Figure 1, Table S3) in a large proportion (42%). The lack of polymorphism at the microsatellite loci in the NE Pacific and NE Atlantic invasive ranges prevented further analyses, in particular of selfing rates and genetic structure among introduced populations.

### 3.2 Genome‐wide diversity in native versus introduced ranges

Stringent filtering steps were applied to avoid false polymorphism detection and resulted in 8,788 polymorphic RAD‐seq loci genotyped in 300 individuals. Polymorphism was successfully detected with this marker panel for both native and introduced populations. The genetic diversity was however much higher in the native range than in the introduced ranges, with 8,580 and 1,988 polymorphic loci, respectively (Table 1, Figure S1). Most of the private polymorphic loci were found in the native range (6,821 loci out of 8,580), but some private loci were also found to be polymorphic only in the introduced ranges (99 in NE Pacific and 58 in NE Atlantic, Table 1, Figure S1). The two introduction ranges also displayed contrasted patterns: nearly twice as many polymorphic loci were detected in the NE Pacific compared to NE Atlantic, with 1,733 and 994 loci, respectively. In addition, most of the polymorphic loci found in the NE Atlantic were present only in two populations, namely Thau (no. 51) and Porto (no. 54; group 2 in Table 1, Figure S1). Outside of these populations, the rest of the NE Atlantic populations (group 1 in Table 1) exhibited only 116 polymorphic loci out of 8,788 (i.e., 1.3% of all retained RAD‐seq loci). A similar pattern was observed in the NE Pacific: only 69 loci (i.e., 0.8% of all study loci) remained polymorphic when considering only the three southern populations of NE Pacific (i.e., Crystal Cove (no. 15), Laguna Beach (no. 16), and Punta Baja (no. 18), Table 1).

**Table 1:** *Sargassum muticum* genetic diversity and estimated selfing rates obtained with Rad‐seq loci across populations in the study native range (Japan) and the two introduced ranges (North East Pacific (NEP) and North East Atlantic (NEA))

	Area	*N*_indiv_	*N*_loc_	*N*_Priv_	*N*_ind‐high_	*H*_area_	*H*_s‐mean_	*s*_g2‐mean_
Native range		78	8,580	6,821	60	0.364	0.161	0.765
Introduced ranges	NEP and NEA	222	1,988	208	195	0.037	0.012	0.251
	NEP only	87	1,733	99	77	0.043	0.017	0.261
	North NEP	39	1,583	98	29	0.057	0.033	0.449
	South NEP	38	69	1	48	0.001	0.001	0.073
	NEA only	135	994	58	118	0.030	0.008	0.244
	Group 1	104	116	5	87	0.001	0.001	0.123
	Group 2 (Thau and Porto)	31	913	52	31	0.041	0.033	0.669

Note

*N*_indiv_, *N*_loc_, *N*_Priv_, *N*_ind‐ high_, *H*_area_, *H*_s‐mean_, and *s*_g2‐mean_ stand for the number of individuals, the number of polymorphic loci, the number of polymorphic loci private to the area, the number of individuals used for the population analyses (i.e., high quality RAD sequencing, with <10% missing data), the gene diversity computed over the whole area, the average value for gene diversity heterozygosity, and selfing rate estimates at population level, respectively. Two groups have been defined in the NE Pacific and NE Atlantic based on genetic structure analyses: (a) in the NE Pacific, a northern and a southern group were distinguished, and (b) in the NE Atlantic, group 1 was composed of all the NE Atlantic populations except Thau and Porto, the two latter forming a second group.

**Figure 2:**
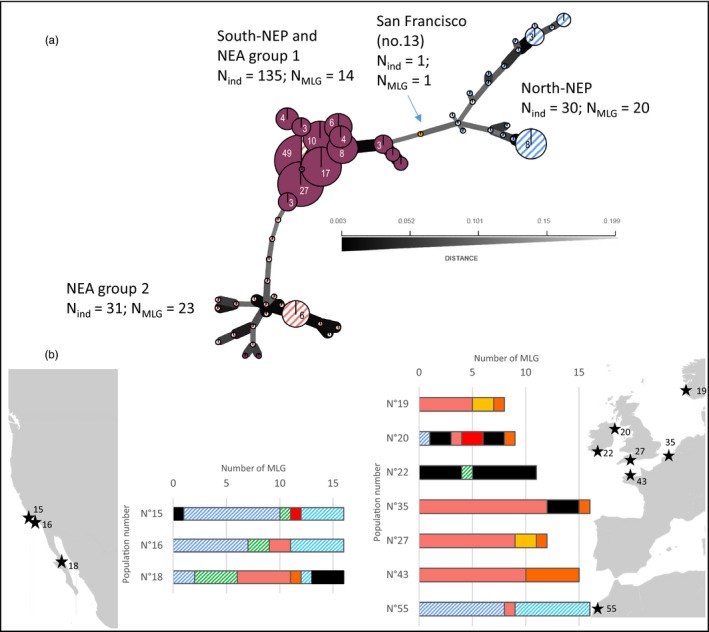
Distribution of *Sargassum muticum* multi‐locus genotypes (MLGs) obtained with RAD‐seq loci in the introduction ranges. (a) Minimum spanning network on multi‐locus genotypes (MLGs) detected in the introduced ranges, based on the number of pairwise allelic differences between MLGs, with gray shading of branch inversely proportional to the distance between MLGs, and (b) population level distributions of the 14 MLGs found in the southern NE Pacific and NE Atlantic Group 1 (i.e., NE Atlantic excluding populations no.51 (Thau) & 54 (Porto)). Population private haplotypes are colored in black

Gene diversity (expected heterozygosity within populations; Hs) ranged from 0.001 to 0.242 with the highest values observed in the native range (Table S5). Averaged over populations or computed at a regional scale (across populations), gene diversity values in the native range were more than 10 times higher than in the introduction range (Table 1). The diversity decrease was particularly strong when comparing the study native range with the NE Atlantic group 1 (all NE Atlantic populations, except Thau (no. 51) and Porto (no. 54), see below) with values of 0.363 and 0.001, respectively (Table 1). The number of multi‐locus genotypes (MLGs) was congruent with the level of genetic diversity, and variable among the different areas, in particular across introduction ranges (Figure 2a). Using poppr, MLGs were found to be clustered in three different lineages (Figure 2a) separating the most northern populations of the NE Pacific (no. 10, 11, 12 in Figure 1), the southern populations of the NE Pacific (no. 15, 16, 18), and NE Atlantic, except for Thau (no. 51) and Porto (no. 54), (referred to as NE Atlantic—Group 1) and finally a lineage with Thau and Porto only (NE Atlantic—Group 2). More in‐depth investigation of the distribution of the 14 MLGs found in the southern NE Pacific and NE Atlantic group 1 revealed subtle differences between the two areas (Figure 2b). First, apart from Kircubbin (no. 20 in Figure 1), the number of MLGs was lower in populations from the NE Atlantic compared with the southern NE Pacific, with one MLG at high frequency in NE Atlantic populations from the Channel and the North Sea (populations no. 43, 27, 35, and 19). One MLG found to be private to one particular NE Atlantic population (i.e., the MLG colored in yellow in population no.20—Kircubbin in Figure 2b) was also found in the southern NE Pacific populations. Interestingly, the MLG distribution found in the recently reported populations of Sidi Bouzid (no. 55) is more similar to the one observed in the southern NE Pacific than in the other NE Atlantic populations (Figure 2b).

Population selfing rates were also variable both among and within geographic ranges. The average value across populations was more than three times higher in the native range (*s* = 0.765) as compared to either the NE Pacific (*s* = 0.261) or the NE Atlantic (*s* = 0.244) introduction ranges (Table 1). In the native range, all the populations exhibited 50% or more selfing, with the highest values obtained in Arida (no. 5 in Figure 1) and Anan (no. 6), potentially strictly selfing populations (*s* > 0.98; Table S5). In the introduced ranges, only five populations located in the northern part of the NE Pacific (no. 10, 12) and in the NE Atlantic (19, 43, 51 and 54) exhibited selfing rate >0.5, yet for the populations no. 12 and 43, the confidence interval is including zero. All the other populations presented low selfing rates, with values not significantly different from zero (Table S5). Altogether, all populations from the southern NE Pacific and five out of seven NE Atlantic populations appeared to be preferentially outcrossing, and at significantly higher rates than in the native range (Wilcoxon rank sum test, *p* = .004).

### 3.3 Genetic structure revealed by RAD‐seq loci

**Figure 3:**
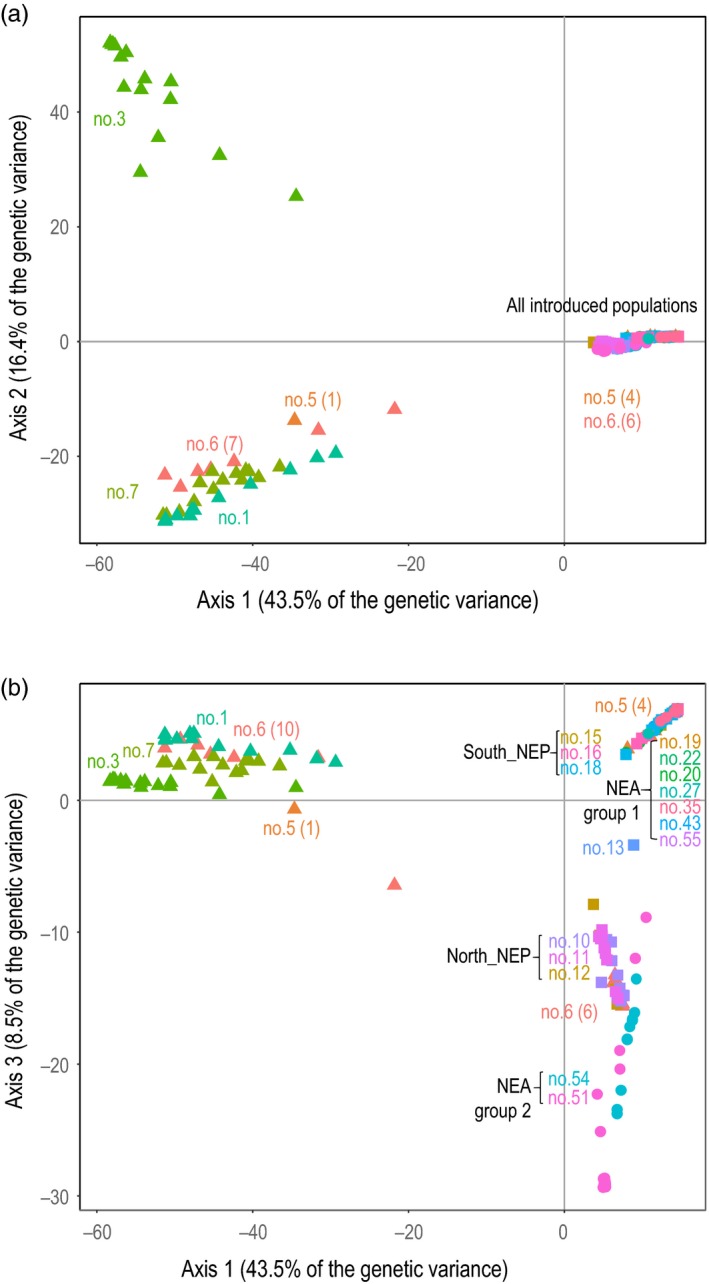
Principal component analysis (PCA), using the RAD‐seq dataset, showing individuals coordinates on the first two components (a) and on the first and third component (b). Colors refer to different regions and populations

A principal component analysis carried out on a set of 256 individuals with <50% of missing data each (leading to 10% of missing data in the whole dataset) revealed genetic differentiation among and within the three areas (Figure 3). The first two principal components, accounting for 43.5 and 16.4% of the genetic variance, discriminated (a) the introduced populations and a few native individuals (Anan (no. 6, *N* = 6), Arida (no. 5, *N* = 4)) from the native populations and (b) the Kashikojima population (no. 3) from the rest of the native populations (Figure 3a). The third principal component (8.5%) revealed differentiation within the introduced range with two clearly distinguished groups (Figure 3b). Interestingly, each group was composed of individuals from both NE Pacific and NE Atlantic: (a) individuals from the northern part of the NE Pacific, namely San Juan Island (no. 10), Tatoosh (no. 11), and Coos Bay (no. 12) grouped with NE Atlantic individuals from Thau (no. 51) and Porto (no. 54; i.e., NEA‐ group 2 in Figure 3b) and (b) the southern populations of the NE Pacific (no. 15, 16, and 18) grouped with the rest of the NE Atlantic localities (NEA group 1 in Figure 3b). The individual from San Francisco Pier 39 (no. 13) appeared at an intermediate position along axis 3 between these two large groups.

**Figure 4:**
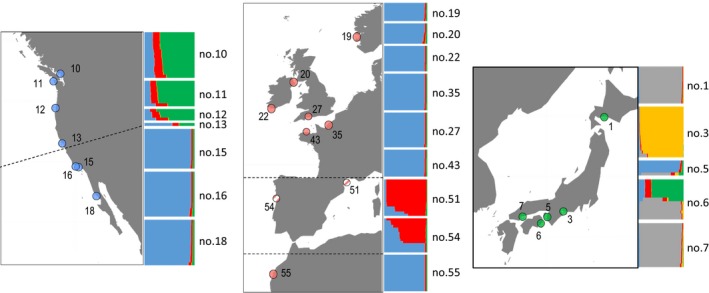
Bayesian clustering analyses (InStruct software, *K* = 5) in native and introduced ranges of *Sargassum muticum,* with 8,788 RAD‐seq loci. Each individual is represented by a vertical line divided into colored segments, the length of which indicates the individual's membership fraction to each of *K* clusters. Individuals are grouped according to their sampling locality. Locality codes correspond to those given in Figure 1 and Table S1

Clustering analyses using InStruct provided congruent results. The lowest deviance information criterion (DIC) over the whole dataset was obtained for *K* = 5. The five clusters defined four different groups of individuals (Figure 4): two genetic groups (corresponding almost exclusively to the yellow and gray cluster) exclusive to the native range, one composed by the northern populations of the NE Pacific and part of the individuals sampled in Anan (no.6), and the last one formed by Arida (no. 5) individuals, the southern populations of the NE Pacific and NE Atlantic populations. The individual of San Francisco Pier 39 (no. 13) displayed a membership pattern intermediate between the northern and the southern NE Pacific populations. Also Thau (no. 51) and Porto (no. 54) displayed a particular pattern with many individuals showing a strong membership to a particular cluster (shown in red in Figure 4), sometimes admixed in variable proportion with the blue cluster. Note, however, that other individuals in Porto (no.54) shared a genetic background 100% similar to the genetic background found in the other NE Atlantic populations. The same clustering was observed by running independent analyses with samples from the introduced ranges only (Figure S2B). Running independent analysis with samples from the native range however allowed to discriminate Muroran (no. 1) from the other native populations, in particular Hiroshima (no. 7, Figure S2A).

Pairwise Fst values and associated dendogram (Figure S3) supported the main findings and population clustering obtained with both PCA and model‐based clustering analyses presented above.

## 4 DISCUSSION

Despite its status of an emblematic worldwide invader, only one previous study based on nuclear, plastidial, and mitochondrial sequencing (Cheang et al., 2010) attempted to elucidate the introduction history at the global scale of the brown alga *Sargassum muticum*. The extremely low polymorphism (with only two mitochondrial haplotypes in the whole study) however prevented such investigations. In this study, we aimed at using supposedly polymorphic nuclear markers, namely microsatellite loci, to investigate the population genetic diversity and structure of this worldwide invader. Despite an extensive sampling (c.a. 1,500 specimens, 55 locations) encompassing the native (North East Asia) and introduced ranges (North East Pacific and the North East Atlantic), unexpectedly, no genetic diversity was detected over the two introduced ranges using 14 microsatellite markers. RAD‐seq loci, however, revealed 1,988 polymorphic loci across the two introduction ranges, compared with 8,580 in the studied part of the native range. These results and their implication on our understanding of the introduction processes and success of the study species are discussed in turn below, notably regarding what has been documented in other marine introduced species.

### 4.1 A poorly diverse but successful introduced species

Our first attempt at elucidating the invasion history of *S. muticum* consisted in gathering microsatellite data on an extensive sampling covering the current latitudinal scale of its introduction ranges, and including native samples from Japan, an area where the introduced populations were presumably coming from (Engelen et al., 2015). In the native range, the genetic diversity was low within but highly structured among populations, a pattern expected and often documented in partial selfing species (Obbard, Harris, & Pannell, 2006; Viard, Justy, & Jarne, 1997). However, the most striking result was the absence of genetic diversity across 14 microsatellite markers over more than 1,000 samples from both introduction ranges (Figure 1). This lack of diversity contrasts sharply with other seaweed cases: for instance, a recent study by Guzinski et al. (2018) showed that a dozen microsatellites provided roughly similar results as several thousand SNPs obtained from ddRAD sequencing to analyze the connectivity and adaptation processes in the introduced seaweed *U. pinnatifida*, at a regional scale. So far, introduced populations of sexually reproducing marine invaders have never been shown to be monomorphic at microsatellites or similar type of highly variable markers. Monomorphism has been reported with either low‐resolution markers, including *S. muticum* with sequencing data from mitochondrial markers (Cheang et al., 2010), or in clonal organisms (e.g., the androgenetic *Corbicula* clams (Pigneur et al., 2014); see other examples and references in Roman and Darling (2007)). The partially clonal green algae *Caulerpa taxifolia* is also well known for its monomorphism in the introduced Mediterranean populations following clonal spread from aquarium release, but it also exhibits higher genetic diversity in a second introduction area (New South Wales, Australia) which might have originated from multiple introduction events and vectors (Arnaud‐Haond et al., 2017).

To investigate the magnitude of genetic diversity loss in the introduced ranges in more detail, we then carried out a genome‐wide analysis using ddRAD sequencing, a technique helpful to get thousands of markers through a reduced representation of the genome. In the native range, measures of genetic diversity and structure, obtained with 8,580 polymorphic RAD‐seq loci, were consistent with the patterns observed with the microsatellite loci, validating the de novo parameters used in the analysis. For instance, population pairwise Fst estimates computed with RAD‐seq loci (Figure S3) and microsatellites (Table S4) in the native range were significantly correlated (Mantel test, *p* = .006). However, polymorphism was also detected in the introduced range with RAD‐seq loci, even though the genetic diversity was much lower than in the native range. As expected following a founder event (Nei, Maruyama, & Chakraborty, 1975), lower values for both the number of polymorphic loci and the gene diversity were observed in the introduction range. At both regional and population scales, on average, a ten‐fold decrease in gene diversity was observed, and per region four to 70 times less polymorphic loci (Table 1). The loss of genetic polymorphism was particularly strong in the NE Atlantic group 1 and the three southern populations of the NE Pacific, two groups genetically distinguished from the other introduced populations. This pattern is not explained by the number of individuals analyzed; for instance, Hs is 0.001 for the 87 individuals NE Atlantic group 1 (ranging from Norway to Morocco), a value to be compared with 0.161 for the 60 Japanese individuals.

Such low polymorphisms are in strong contrast to RAD‐seq data obtained in other marine invaders. In the seaweed *U. pinnatifida*, native to Asia and introduced in NE Atlantic, Guzinski et al. (2018) examined populations established in Brittany (France, NE Atlantic) since the 1980s using ddRAD sequencing (and a similar protocol as in our study) and retained more than 10,000 polymorphic loci. Besides the present work, this study of *U. pinnatifida* provides to our knowledge the only RAD‐seq‐based analysis of introduced seaweeds published so far. However, other studies on marine introduced animals using the same technique have been carried out. For instance, Jeffery, DiBacco, Van Wyngaarden, et al. (2017) found 9,137 polymorphic loci in the Canadian introduced range of the crab *Carcinus maenas*. Similarly, in the mussel *Mytilus galloprovincialis*, Saarman and Pogson (2015) examined more than 1,137 polymorphic loci in the central Californian introduced range alone. Therefore, by comparison with these studies, if not monomorphic, introduced populations of *S. muticum* still exhibited a drastic reduction of the genetic diversity, which might be due to strong selective events and/or a severe bottleneck during the early stages of the introduction.

Irrespective of the markers used, *S. muticum* thus shows a remarkable genetic diversity pattern when compared to the other marine invaders documented (Rius et al., 2015; Viard et al., 2016). The vast majority of marine introductions are characterized by repeated introductions and/or high propagule pressure that accumulate genetic diversity over time in introduced populations. This large genetic diversity is expected to provide a basis on which selection could operate, to contain genotypes that are preadapted to the local environment, or to favor evolutionary novelties through recombination between distinct genetic lineages (Bock et al., 2015; Rius & Darling, 2014; Rius et al., 2015; Viard et al., 2016). Only in a few cases, reduced genetic diversity has been shown in sexually reproducing introduced marine populations. For example, this is the case of the gastropod *Rapana Venosa*: Chandler, McDowell, and Graves (2008) showed a lack of genetic diversity at two combined mitochondrial gene regions (COI and ND2), with a single haplotype in the introduced ranges, compared with 110 in the native range. This strong genetic bottleneck was later confirmed with microsatellite markers by Xue et al. (2018) with introduced populations showing on average 6.7 alleles to be compared with 22.9 in the native range. Reduced genetic diversity was also reported in *Corella eumyota* in its European introduction range (Dupont, Viard, David, & Bishop, 2007), with <60% of polymorphic loci, but its introduction was presumably recent and its extent limited. Also, like *S. muticum*, *C. eumyota* is a partial selfer. Selfing, known in both terrestrial plants and animals to facilitate colonization of new habitats and rapid growth of self‐sustained populations (Baker, 1967; Pannell, 2015; Pannell & Barrett, 1998), certainly played a significant role in the invasion success *S. muticum*, a feature discussed hereafter.

### 4.2 Revisiting the introduction history and pathways of *Sargassum muticum*

Using ddRAD sequencing data, three distinct genetic entities were detected across all introduced ranges, but there was limited geographic coherence in genetic patterns observed using InStruct (Figure 4), PCA (Figure 3), and the minimum spanning network on multi‐locus genotypes (Figure 2). Northern populations of the NE Pacific and most of the individuals found in two NE Atlantic populations (Thau (no. 51) and Porto (no. 54)) each displayed a distinct genetic background, presumably pointing at two distinct genetic sources. Southern populations of the NE Pacific and the rest of European populations, however, shared a common and distinct genetic background, suggesting a common source different from the two previous ones (Figure 4).

The genetic patterns are also poorly coherent with the known (i.e., reported) history of introduction. When considering the dates of first report of *S. muticum* along the North American coasts (reviewed in Engelen et al. (2015)), the eldest and first observation of the alga outside of its native range was in the Puget Sound, in British Columbia (Canada) in the northern NE Pacific, in the early 1940s. The following records, for the next 30 years, were from the same or nearby areas. Reports of established populations of the species in south NE Pacific were made only in the 1960s in California and, later, along the Californian coastline down to Mexico. Altogether, these reports suggested a stepwise southward expansion of the species from the Puget Sound to California. The late arrival in California could be explained by the existence of the biogeographic boundary, near Point Conception, separating the Californian and Oregonian biogeographic provinces (Burton, 1998). This area is characterized by upwelling and variable surface currents as well as heterogeneous coastline topography, including a long stretch of sandy coasts, which constitute potential environmental barriers to natural dispersal (Dawson, 2001; Hohenlohe, 2004), in particular for *S. muticum* colonizing rocky shores. These barriers might have slowed down the southward expansion until large populations have been established in the northern NE Pacific. However, in contrast to this southward expansion scenario, our results strongly suggest that the establishment of *S. muticum* along the NE Pacific coasts is due to two independent introduction events and presumably from different origins. It is noteworthy that under this “two‐introduction” scenario, the biogeographic boundary could have also played a major role in preventing exchanges between populations originating from the two distinct northern and southern introduction events.

One explanation for the discrepancy between the presumed introduction pathway (i.e., southward expansion from the primary introduction in the Puget Sound ending in California in the 1960s) and the genetically based scenario (i.e., two independent events) could be that dates of the first report in California poorly represent the actual dates of the first establishment of the species. A “Report from California oyster culture” (Barrett, 1963) indeed reported oyster imports from Japan in several Californian Bays as soon as the 1930s and with successful planting of Pacific oyster seed oysters. *S. muticum* could, therefore, have been introduced accidentally in the southern NE Pacific during these deliberate oyster imports well before its date of first report in this area. One should be cautious on considering the date of first observation to infer the introduction history of NIS, especially in the marine environment where field surveys are sometimes hard to carry out or with insufficient observation pressure. In addition, they could largely be biased by the recording interest that can be triggered by the discovery of a new NIS in a specific area. Invasion histories are typically challenging to reconstruct with confidence, certainly when historical information is sparse and genetic data of the initial stages of the invasion process are not available (Möst et al., 2015). This is exemplified by *Codium fragile*, a case study for which the genetic analysis of herbaria specimens showed that its introduction occurred well before (nearly 100 years) the reported date of introduction (Provan, Booth, Todd, Beatty, & Maggs, 2008). These putative biases should be taken into account in standardized monitoring and surveillance protocol of (new) NIS, which should attempt to include as many species as possible, and not only focus on high‐priority species. To reach this challenging objective, new molecular methods, for instance based on metabarcoding of environmental DNA, might be particularly relevant to use to support surveillance programs (Darling et al., 2017).

Regarding the introduction in the NE Atlantic, previous assumptions based on surveys (Engelen et al., 2015) or mitochondrial data (Cheang et al., 2010) suggested that the introduction in Europe is a secondary introduction from North America, presumably with imports of oysters from the northern part of the NE Pacific in the 1970s, rather than a distinct introduction from the native range. This hypothesis was reinforced by the existence of similar scenarios for other marine introductions linked to oyster translocations (e.g., the gastropod *Ocinebrellus inornatus*; Martel et al., 2004). Under this scenario, however, the epicenter of secondary seeding of NIS in Europe should be localized in the Puget Sound in North America. Conversely, populations in Europe were found to share a common genetic background with the southern NE Pacific populations. Moreover, this similarity is even more pronounced for the African population (Sidi Bouzid, no. 54) which is more similar to Californian populations than to any other NE Atlantic populations based on MLGs (Figure 2), which may suggest an independent introduction event from California. Given the high genetic structure among populations and the occurrence of numerous multi‐locus genotypes in the native range, it is unlikely that two independent introduction events in two continents which occurred 40 (i.e., under the scenario of cryptic introduction in California in the 1930s) or even 10 years apart (i.e., using the first report date in the 1960s) originated from the same sources with the same genotypes introduced twice. Thus, our data support a scenario of a secondary introduction from the NE Pacific to NE Atlantic but from a distinct source as previously assumed (the South NE Pacific instead of North NE Pacific).

Determining the source of the introductions in the different parts of the present‐day distribution range of *S. muticum* was not the scope of this study. It would have required extensive sampling in the native range. Here we focused on Japan, including areas which were presumed to have been putative sources (major oyster farming sites). We thus cannot ascertain the origin of any of the genetic entities revealed by the RAD‐seq data. It is, however, noteworthy that part of the Anan individuals shares an identical genetic background with individuals in the southern NE Pacific and the NE Atlantic (Group 1; Figure 4). Anan may be either the source of the introduction in these parts of the NE Pacific and Atlantic but could also have been a recipient population from other populations in the native range as oyster transfers can also occur among Japanese oyster farms.

All of these facts form a body of evidence suggesting that 1) three independent introductions occurred at a global scale, 2) northern and southern populations of *S muticum* in NE Pacific originated from two distinct introduction events, and 3) the southern populations could have seeded most of the European populations. Massive sampling in the native range might be helpful to investigate these scenarios, although, with longer time elapsed since the introduction, the likelihood to identify the sources is fading rapidly in highly genetically structured species (Geller, Darling, & Carlton, 2010) such as our study species. This study also adds to previous genetic studies which showed unreported (cryptic) introductions in marine environment (Geller et al., 2010; Rius et al., 2015), thus revealing a necessary control of the introduction vectors and pathways, here targeting in particular aquaculture trade.

### 4.3 Potential admixture between lineages originating from distinct introduction events

Besides the presence of two distinct introduction events characterized by different genetic background, our genome‐wide survey also suggests that admixture between different genetic lineages might have occurred in each of the two introduction ranges. In the NE Pacific, we indeed showed a clear genetic distinctiveness between the northern and southern groups, separated by a well‐known natural biogeographic barrier. However, both InStruct (Figure 4) and PCA (Figure 3) analyses revealed an intermediate genetic background in the individuals sampled in San Francisco (no. 13), and, interestingly, the MLG found in San Francisco displayed an intermediate position on the minimum spanning network between the northern and southern groups of MLGs (Figure 2). Note that carrying out a PCA with the less conservative dataset allowed to retain the second individual sampled in San Francisco, which also displayed an intermediate genetic background (data not shown). The very limited sample size prevented further analyses, and definite conclusion regarding the status of this introduced population. However, it is noteworthy that the two individuals were sampled in a marina: human‐mediated transports by leisure or commercial boats can facilitate secondary contact, by transporting individuals across natural barriers, and thus promote admixture between distinct genetic origins. A similar situation of admixture between two introduced genetic lineages, following transport by anthropogenic activities, has been reported in one introduced population of the green crab *Carcinus maenas* in Newfoundland (Jeffery, DiBacco, Wringe, et al. (2017) and references therein).

In the NE Atlantic introduction range, InStruct analyses also revealed individuals with intermediate memberships to two distinct genetic clusters (pictured in red and blue in Figure 4) in Thau (no. 51), and particularly in Porto (no. 54), again suggesting admixture between distinct lineages. Interestingly, in Porto, several individuals also displayed the typical genetic background found in the other populations distributed along the NE Atlantic coast. We might hypothesize that a distinct lineage had been introduced and successfully established in the Mediterranean Sea (here illustrated by the Thau population (no. 51) in Figure 4), for instance favored by the local environmental conditions (e.g., warmer seawater). The subsequent introduction of this lineage in Porto might have led to admixture between the two lineages. In the American introduced range of *C. maenas* hybridization between two lineages independently and successively introduced, each of them presumably adapted to different thermal conditions had been shown (Jeffery, DiBacco, Wringe, et al., 2017). A similar situation might occur along the Portuguese coasts. Admixture between distinct evolutionary lineages that had been independently introduced over time is a process that might enhance the colonization success of introduced species (Prentis, Wilson, Dormontt, Richardson, & Lowe, 2008), although evidence is still scarce in marine environments (Rius & Darling, 2014). Studying a larger number of individuals, and, more importantly, of populations along the Portuguese coast could shed light on the extent of this hypothetical admixture scenario in the NE Atlantic and of the role of the environment.

### 4.4 Could selfing rate variation be a key factor to succeed from one introduction phase to the next?

Despite severe founder events, and associated loss of genetic diversity, *S. muticum* successfully colonized two oceans and 11 countries in <70 years and continues to spread (e.g., first report in Morocco in 2012). As compared to other sexually reproducing marine invaders, and regarding the absence of genetic diversity in introduced populations, phenotypic plasticity or epigenetic factors are probably important mechanisms by which the species could accommodate a broad range of environmental conditions (Engelen et al., 2015), thus favoring a good match between local environmental conditions and ecological requirements of the introduced species. In addition, *S. muticum* displays reproductive properties that may limit demographic stochasticity: (a) high selfing rates, usually associated with limited inbreeding depression and (b) high fertility (Engelen et al., 2015). A single or a few individuals can seed a rapidly growing population. As shown in terrestrial invasive plants (e.g., Hao, Qiang, Chrobock, van Kleunen, & Liu, 2011; Rambuda & Johnson, 2004) and in a few algal species (e.g., the invasive red alga *Gracilaria vermiculophylla*, Krueger‐Hadfield et al. (2016)), these features could have played a crucial role at various stages of the introduction process of *S. muticum*, notably the early stages (i.e., reproductive assurance hypothesis). We first hypothesized that selfing rates should be higher in introduced populations as compared to native ones, but the RAD‐seq dataset actually documented the opposite (Table 1). We also observed variable levels of selfing rates among introduced populations (Table 1): almost all the most recently introduced populations, namely populations from NE Atlantic—Group 1 (introduced after the 1970s), displayed (a) barely any gene diversity and (b) a mating system shift with decreased selfing rates, as compared to the native range and other earlier introduced populations (e.g., northern NE Pacific in the 1940s). The same pattern is observed in southern NE Pacific although more controversial, as these populations had been reported after the 1960s but our genetic data cast doubt on this scenario. The highest selfing rates were observed in northern NE Pacific populations reported there from the early 1940s, thus established for more than 70 years.

These observations suggest a shift in selfing rates, increasing over time since the introduction. Early populations could have consisted of a limited number of lineages, either preadapted to local conditions and plastic enough to acclimate to the new conditions or newly evolved from outcrossing events occurring between distinct recently introduced genetic lineages. Low selfing rates in the first stage of the introduction may indeed provide opportunities for the evolution of new genotypic combinations through recombination, in newly and poorly diverse introduced populations. This might explain the larger amount of private MLGs reported in the NE Atlantic and southern NE Pacific populations (Figure 2), which accounts for half of the total MLGs found in this area. Then, evolution toward increased selfing rates might contribute not only to enhance reproductive assurance at the front of the expansion (i.e., similar to range margins) but also to accelerate the fixation of an advantageous genotype during the establishment stage, thus sustaining local adaptation and expansion to similar habitats (Jarne & Charlesworth, 1993; Pannell et al., 2015). Mature specimens, sometimes carrying fertilized eggs, can spread over large distance through drifting thalli (Fletcher & Fletcher, 1975). If drifting thalli are sexually mature, they may colonize favorable habitats, especially considering that with selfing, only one individual can seed a new population and, that way, spread its genotype efficiently. Genetic diversity has been hypothesized to be an important correlate of marine invasion success (Rius et al., 2015; Roman & Darling, 2007; Viard et al., 2016). The demonstrated strongly reduced genetic variation, indicative of strong founding events and/or selective process, however did not inhibit the sustainable establishment and rapid spread of *S. muticum*. Examining herbarium specimens dating from the first reports would have been extremely valuable to determine whether many lineages were initially introduced or whether some new MLGs evolved and increased in frequency over time, and, if this is the case, which ones were the “winner” of the introduction lottery. We attempted to use such herbarium specimens. Unfortunately, we could not get enough good quality DNA, even for genotyping microsatellites, to examine these specimens. Whatever the mechanisms initially involved, the ability to self and restore populations with a single or a few individuals seems to be one of the most important factors explaining the rapid establishment of *S. muticum* over large scales. For preventing its future spread, surveillance programs should thus pay particular attention on drifting individuals, which, if fertile, might seed a new population: such drifting individuals should be rapidly removed when found in new places. In addition, campaigns removing *S. muticum* should take extreme care not to produce fertile drift material. More generally, our results suggest that self‐compatible marine NIS should be particularly well monitored, as already documented in terrestrial introduced plants (e.g., Milbau & Stout, 2008).

## 5 CONCLUSIONS

The spread of *S. muticum* in its introduction range has been very well documented, and hundreds of studies have been dedicated to this seaweed. Yet, the genetic data obtained in this study, for instance, suggesting previously unreported introductions in both the NE Pacific and NE Atlantic, show that we actually know little about the invasion history of this notorious invasive seaweed. *S. muticum* turned out to be a unique example of a successful nonclonal (i.e., sexually reproducing) marine introduced species which exhibits almost no genome‐wide genetic variation over most of its circumglobal introduction range. Our study also demonstrates that RAD sequencing is a powerful tool to detect genetic variation for nonmodel species, in the case of extremely reduced genetic diversity, following severe founder events. Our result does not mean that genetic diversity is not important for the long‐term establishment of invasive species but emphasizes the need to be extremely cautious in interpreting neutral genetic diversity as an indicator of adaptive genetic diversity and potential success of an invader. Epigenetic processes should be investigated in detail in *S. muticum* as they may also explain the acclimatization of this seaweed to large latitudinal gradients and a diverse set of habitats, without adaptive genetic diversity.

## AUTHOR CONTRIBUTIONS

F.V. conceived the study and analyzed the data. S.L.C., S.B., and C. D‐T conducted the molecular experiments and analyzed the data. A.E. and N.M. contributed to the sampling design and to the sampling. F.V., S.L. C., and C.D.‐T wrote the first draft of this paper. All authors reviewed the manuscript.

## DATA AVAILABILITY

ddRAD sequencing and microsatellite dataset are available as a VCF file and a multi‐locus genotype file, respectively, uploaded on Dryad Digital Repository at https://doi.org/10.5061/dryad.1rj6442 (Le Cam,, Daguin‐Thiébaut, Bouchemousse, & Viard, 2019). In addition, demultiplexed individual fastq files from Illumina Hiseq 2,500 are available under the Bioproject PRJNA549138 on the NCBI portal (Le Cam, Daguin‐Thiébaut, & Viard, 2019).
