# Supplementary material for: A genome‐wide investigation of the worldwide invader Sargassum muticum shows high success albeit (almost) no genetic diversity
Source: Evol Appl. 2019 Aug 14;13(3):500–14. doi: 10.1111/eva.12837 (PMC7045713; doi:10.1111/eva.12837)

Supplementary Information

Table S1. Sampling information for *Sargassum muticum* populations. Populations code (see Figure 1 in the Main Manuscript for geographic location), collectors, sampling year, number of individuals genotyped with microsatellite markers (N_{msat}) and the number of individuals subsampled in the RADseq analyses ($N_{Rad\text{-}Seq}$)

Number	Location	Country	Collector(s)	Date of sampling	N_{msat}	$N_{Rad\text{-}Seq}$
1	Muroran	Japan	K. Kogame	2006	32	16
2	Oshima	Japan	AH. Engelen, J. Boavida, D. Paulo	2007	32	
3	Kashikojima	Japan	AH. Engelen, J. Boavida, D. Paulo	2007	32	16
4	Inhoshima	Japan	AH. Engelen, J. Boavida, D. Paulo, RO Santos	2007	29	
5	Arida	Japan	AH. Engelen, J. Boavida, D. Paulo, RO Santos	2007	32	15
6	Anan	Japan	AH. Engelen, J. Boavida, D. Paulo, RO Santos	2007	31	16
7	Hiroshima	Japan	AH. Engelen, J. Boavida, D. Paulo, RO Santos	2007	32	15
8	Sobol Bay	Russia	A. Skriptsova	2009	11	
9	Craig	USA, AK	D. Garza	2006	1	
10	San Juan Island	USA, WA	K. Britton-Simmons	2006	24	16
11	Tatoosh Island	USA, WA	C. Pfister	2005	18	14
12	Coos Bay	USA, OR	D. Haydar, J. Carlton	2005	24	7
13	San Francisco (Pier 39)	USA,CA	S. Le Cam, S. Bouchemousse	2013		2
14	San Luis Obispo	USA, CA	C. Roe BEll	2006	24	
15	Crystal Cove	USA, CA	J. Smith, S. Murray	2005	24	16
16	Laguna Beach	USA, CA	J. Smith, S. Murray	2005	24	16
17	Enseñada (Playa La Esmeralda)	Mexico	AH Engelen	2010	23	
18	Punta Baja	Mexico	L. Aguilar-Rosas, R. Aguilar-Rosas	2006	24	16
19	Øygarden	Norway	K. Sjøtun, T. Aires	2006-2014	46	14
20	Kircubbin	UK	F. Mineur	2006	22	16
21	Carnson Point	Ireland	C. Golléty	2011	36	
22	Lough Hyne	Ireland	C. Golléty	2011	19	15
23	Widemouth	UK	N. Mieszkowska	2011	32	
24	Newquay	UK	N. Mieszkowska	2011	24	
25	Porthleven	UK	N. Mieszkowska	2011	24	
26	Looe	UK	N. Mieszkowska	2011	24	
27	Wembury	UK	N. Mieszkowska	2011	31	15
28	Langerstone Point	UK	N. Mieszkowska	2011	23	
29	Brixham	UK	N. Mieszkowska	2011	24	
30	Torquay	UK	N. Mieszkowska	2011	24	
31	Lyme Regis	UK	N. Mieszkowska	2011	32	
32	Osmington Mills	UK	N. Mieszkowska	2011	23	
33	Ostende	Belgium	F. Kerckhof	>2000	20	
34	Audresselles	France	F. Gevaert	2011	32	
35	Wimereux	France	F. Gevaert	2011	32	16
36	Blainville sur Mer	France	J.-C. Leclerc	2011	32	
37	Saint-Malo	France	L. Levêque, F. Viard	2011	32	
38	Saint-Cast	France	R. Lasbleiz, F. Riquet	2011	32	
39	Bréhat	France	L. Levêque, F. Viard and colleagues	2009	32	
40	Perros-Guirec	France	L. Levêque, F. Viard	2011	32	
41	Trébeurden	France	L. Levêque, F. Viard	2011	32	
42	Carantec	France	C. Daguin-Thiébaut	2011	32	
43	Roscoff	France	L. Levêque,, T. Aires	2006-2014	45	14
44	Cléder	France	S. Bouchemousse, L. Noel, F. Viard	2011	32	
45	Aber Wrac'h	France	M. Camusat, Y. Fontana	2011	32	
46	Plouzané	France	V. Stiger	2011	32	
47	Camaret	France	L. Levêque, W. Thomas	2011	32	
48	Concarneau	France	S. Bouchemousse, C. Daguin-Thiébaut	2011	32	
49	Ploemeur	France	V. Stiger	2011	32	
50	Quiberon	France	L. Levêque and colleagues	2011	32	
51	Thau (Sète)	France	N. Bierne	2011	32	16
52	La Coruña	Spain	L. Couceiro, A. Geoffroy, S. Krueger-Hadfield, S. Mauger	2011	31	
53	Viana de Castelo (Montedor)	Portugal	L. Couceiro, A. Geoffroy, S. Krueger-Hadfield, S. Mauger	2011	32	
54	Porto (Leça de Palmeira North)	Portugal	L. Couceiro, A. Geoffroy, S. Krueger-Hadfield, S. Mauger	2011	31	16
55	Sidi Bouzid	Morocco	T. Aires	2014	24	16

Table S2. Characteristics of the microsatellite markers developed for *Sargassum muticum*.

Locus code, multiplex code, forward and reverse primers, repeat array, allele size and number of alleles (Nall) over all the studied individuals (N=1500). Accession numbers for the microsatellite sequences: JQ860127-JQ860140.

Locus	multiplex	Forward primer (5'-3')	Reverse primer (5'-3')	repeat array	allele size	Nall
SmC08	A	ACCAACAAGCTTCAGCCTTC	CGTACAGGGTAACGGTCAGG	$(GT)_3T(TG)_7T_4(GATTT)_2$	125, 137	2
SmC24	A	ACTTGCTGTGGTACTTAACGC	TTCTAGTAACGATACGACAACCG	$(TAG)_3(TGG)_5(TG)_2$	96, 99, 102	3
SmD05	A	ATGTGGATTATTATAAGGTTGGGG	AACGGAGTACGGTAGTGTCTGA	$(AC)_{10}$	187, 189, 191	3
SmD10	B	CGACACTATCGTGCAACAGC	CCGAAGAAGGCCGAAATACT	$(CA)_9$	210, 212	2
SmD33	B	ATGATTTTGTCCTTTTGCAGG	GGAGTTCATCGCGGATAGAT	$(CT)_7$	289, 291	2
SmD34	A	ATCAATCATCGCACACTAAGTCA	TGTTTTCTCTATGGCTCATTGTCT	$(CA)_8$	187, 189	2
SmD41	A	TTCCATTTTCCTTGTTCAGC	TGTTGCTGTAGGGATGTGTCA	$(CA)_7$	144, 149	2
SmQ02	B	GCAATCAATCGCTGGTGATA	TTGTCTGGGCTATCCTCTCC	$(ATTG)_7$	78, 87	2
SmQ03	A	ATAGACTCACTCGCTCGTCG	ACCACCCACCCTAATACGAA	$(ATAG)_6$	132, 139	2
SmQ04	B	CTGAGATAGATACGGAATGACAACA	ACCACGGGCTGGGTCTTT	$(TTGA)_6$	118, 130, 138	3
SmT13	B	GAACGGTCATTAGCCGTAGC	TTGTAATATTGTAGCCCTTAGTGGAA	$(ACC)_8$	136, 139	2
SmT15	B	GGTGTCAGAGGGATGATGTAGG	CGCCTACCAGGGTTAAGCTA	$(TGT)_8$	103, 106	2
SmT29	B*	CACGGGCTGAATCTTTGAG	CCTGTAAGGGCCGATGTTAC	$(CAA)_6$	174, 175	2
SmT30	B	CAGTTTGACGCATCACTGCT	GCCTAGGAACGAAGGTGGAC	$(ACC)_6$	173, 176	2

*: locus T29 was amplified separately and co-electrophoresed with the other loci from multiplex B.

Table S3. Genetic diversity and selfing rates, computed for the microsatellite panel, in *Sargassum muticum* populations.

N: number of individuals; n_{all}: mean number of alleles per locus; H_e: gene diversity; N_{MLG} number of multilocus genotypes; MLG_{priv}: number of private multilocus genotypes; D: genotypic diversity. Selfing rate estimates (i) sg2 (test Sg2=0 by 1000 iterations), (ii) s_{ML}: maximum likelihood estimate (95% confidence interval)

		Sampling size	Allelic diversity		Genotypic diversity			Selfing rate estimate	
no.	Locality	N	n_{all}	H_e	N_{MLG}	MLG_{priv}	D	s_{g2} *(P-value)*	s_{ML} (CI 95)
Population									
1	Muroran	32	1.14	0.005	2	2	0.637	0.999 *($<10^{-3}$)*	0.988 *(0.941-0.999)*
2	Oshima	32	1.43	0.096	14	14	0.063	0.785 *(0.072)*	0.799 *(0.000-0.941)*
3	Kashikojima	32	1.43	0.062	8	8	0.710	0.000 *(0.112)*	0.000 *(0.000-0.959)*
4	Inhoshima	29	1.43	0.120	15	13	0.781	0.194 *(0.267)*	0.187 *(0.000-0.823)*
5	Arida	32	1.07	0.009	2	1	0.760	--	--
6	Anan	31	1.64	0.325	5	4	0.904	0.999 *($<10^{-3}$)*	0.992 *(0.988-0.999)*
7	Hiroshima	32	1.36	0.103	10	8	0.121	0.908 *(0.001)*	0.903 *(0.778-0.955)*
8	Sobol Bay	11	1.57	0.137	8	8	0.843	0.764 *(0.045)*	0.745 *(0.000-0.908)*
Region									
1-8	Asia (native)	231	2.21	0.364	60	59	0.921	NA	NA
9-17	NE Pacific	186	1	0	1	0	0	NA	NA
18-54	NE Atlantic	1083	1	0	1	0	0	NA	NA

Table S4. Theta-estimates of Fst, following Weir & Cockerham (1984), obtained with microsatellite loci, between pairs of Asian populations of *Sargassum muticum*.

All theta values are significantly different from zero (permutation test; 2000 perm.; Genetix software). The null hypothesis of similar distribution of allelic frequencies between populations was also tested with an exact test implemented in Genepop v4.0: the null hypothesis was rejected for all pairwise comparison ($P<10^{-5}$).

Theta	Oshima	Kashikojima	Inhoshima	Arida	Anan	Hiroshima	Sobol Bay
Muroran	0.858	0.961	0.878	0.987	0.767	0.863	0.879
Oshima		0.844	0.533	0.791	0.524	0.592	0.710
Kashikojima			0.820	0.929	0.500	0.856	0.863
Inhoshima				0.358	0.369	0.196	0.638
Arida					0.452	0.485	0.847
Anan						0.472	0.521
Hiroshima							0.583

Table S5. ***Sargassum muticum*** **genetic diversity and estimated selfing rates obtained with Rad seq loci in introduced and native populations.**

N_{indiv}, N_{loc}, $N_{ind\ high}$, H_s and s_{g2} stand for the number of individuals studied, the number of polymorphic loci, the number of individuals used for the population analyses (i.e., high quality Rad-sequencing, with less than 10% missing data), the gene diversity and selfing rate estimates (maximum likelihood estimate with 95% confidence interval). Population number (no) refer to numbers in Fig. 1 in the main manuscript.

	Pop Name	no.	N_{ind}	N_{loc}	N_{ind_High}	H_s	s_{g2} (CI 95)
**Native range**							
	Muroran	1	16	2035	12	0.091	0.595 (0.307-0.693)
	Kashikojima	3	16	3843	16	0.120	0.767 (0.543-0.823)
	Arida	5	15	3626	5	0.172	0.993 (NaN-0.995)
	Anan	6	16	4354	13	0.242	0.976 (0.430-0.983)
	Hiroshima	7	15	4626	14	0.182	0.495 (0.161-0.617)
**Introduced range**							
	**North East Pacific – northern part**						
	San Juan Isl.	10	16	1315	16	0.043	0.730 (0.558-0.811)
	Tatoosh	11	14	454	9	0.017	0.000 (0.000-0.034)
	Coos Bay	12	7	574	4	0.040	0.793 (0.000-0.914)
	San Fransisco	13	2	273	1	0.000	NA
	**North East Pacific – southern part**						
	Crystal Cove	15	16	37	16	0.001	0.315 (0.000-0.472
	Punta Baja	18	16	31	16	0.001	0.000 (NaN-0.335)
	Laguna Beach	16	16	30	16	0.001	0.266 (0.000-0.471)
	**North East Atlantic – Group 1**						
	Øygarden	19	14	46	8	0.001	0.535 (0.153-0.729)
	Kircubbin	20	15	47	9	0.001	0.000 (0.000-0.185)
	Lough Hyne	22	14	32	11	0.001	0.245 (0.000-0.450)
	Wimereux	35	16	22	16	0.001	0.000 (0.000-0.236)
	Wembury	27	15	22	15	0.001	0.261 (0.000-0.436)
	Roscoff	43	14	45	12	0.001	0.693 (0.000-0.780)
	Sidi Bouzid	55	16	15	16	0.001	0.000 (0.000-0.000)
	**North East Atlantic – Group 2**						
	Porto	54	15	867	15	0.046	0.857 (0.538-0.918)
	Thau	51	16	848	16	0.021	0.812 (0.171-0.852)

Fig. S1. Venn diagram showing the number of polymorphic Rad-Seq loci shared among different geographic entities in the native and introduced ranges of *Sargassum muticum*.

NEP and NEA stand for the two introduction ranges, namely North East Pacific and North East Atlantic, respectively.

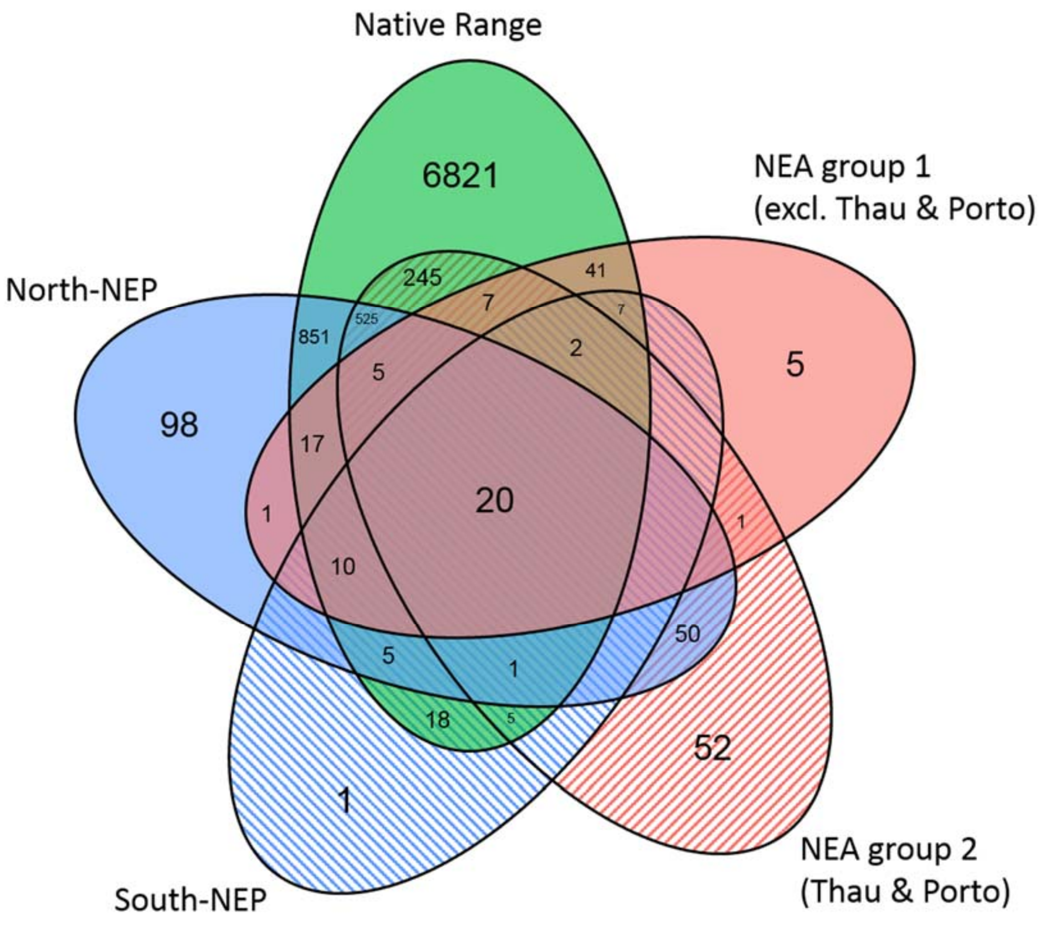

Figure S2. Bayesian clustering results and individual memberships, obtained with InStruct analyses carried out separately (A) for the native range (K=5) and (B) the introduced range (K=3)

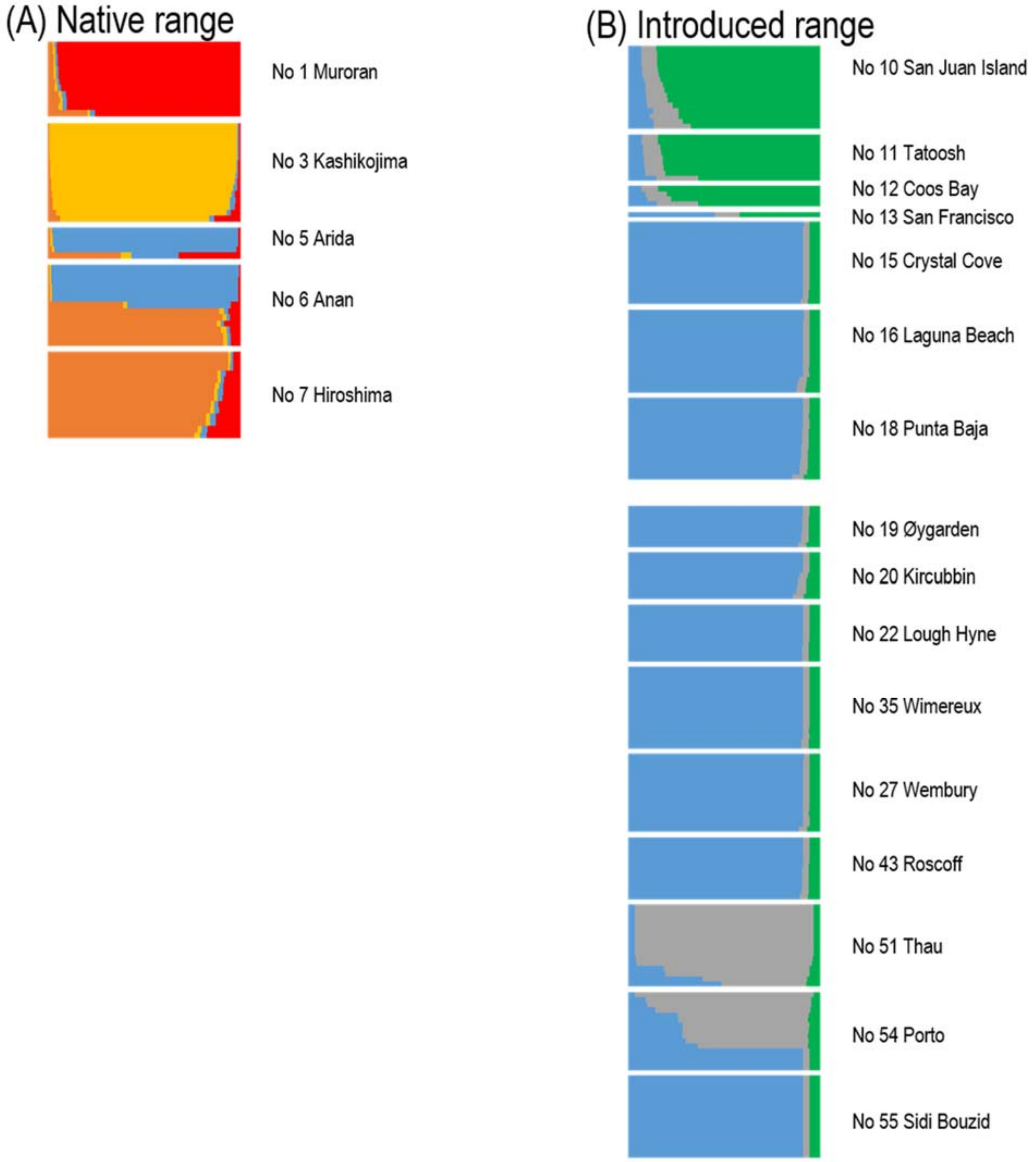

Fig S3. Heatmap of *Sargassum muticum* population pairwise Fst estimates (Theta estimate of Weir & Cockerham (1984)) reordered according to the Fst-based dendogram. Color key is indicated in legend together with the distribution of the value (density). The geographic area of the populations is color-coded below the dendogram (Green: Native range, Blue: introduced range in North East Pacific and Pink: introduced range in North East Atlantic)

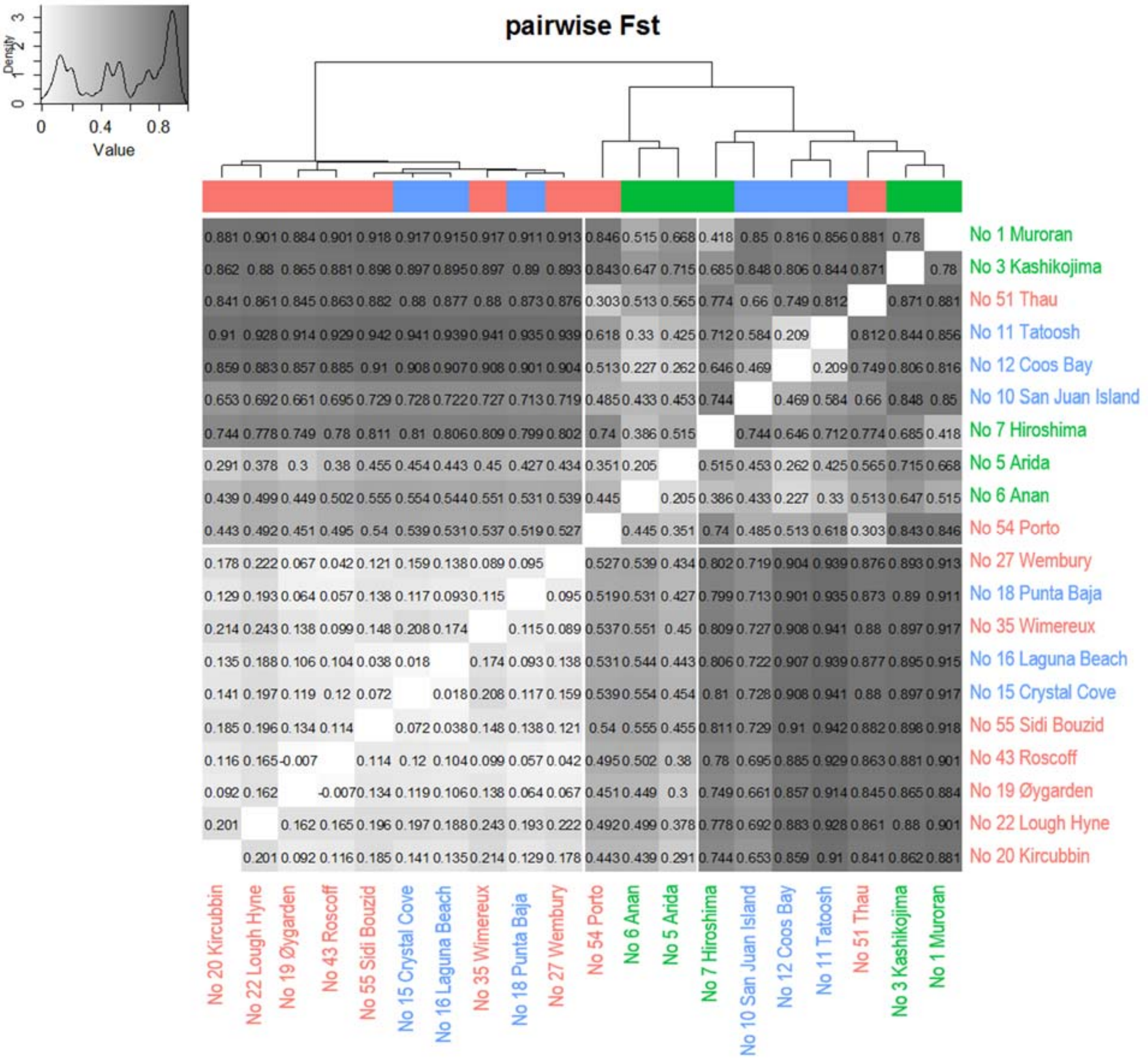

Supplement: Supplementary file 1 [file EVA-13-500-s001.pdf]
